# The effectiveness of health appraisal processes currently in addressing health and wellbeing during spatial plan appraisal: a systematic review

**DOI:** 10.1186/1471-2458-11-889

**Published:** 2011-11-24

**Authors:** Selena Gray, Laurence Carmichael, Hugh Barton, Julie Mytton, Helen Lease, Jennifer Joynt

**Affiliations:** 1 Department of Health and Applied Social Studies, Faculty of Health and Life Sciences, University of the West of England, Room 2B06, Glenside Campus, Blackberry Hill, Stapleton, BS16 1DD Bristol, UK; 2 WHO Collaborating Centre for Healthy Cities and Urban Policy, Faculty of Environment and Technology, University of the West of England, Bristol, UK

## Abstract

**Background:**

Spatial planning affects the built environment, which in turn has the potential to have a significant impact on health, for good or ill. One way of ensuring that spatial plans take due account of health is through the inclusion of health considerations in the statutory and non statutory appraisal processes linked to plan-making processes.

**Methods:**

A systematic review to identify evaluation studies of appraisals or assessments of plans where health issues were considered from 1987 to 2010.

**Results:**

A total of 6161 citations were identified: 6069 from electronic databases, 57 fromwebsite searches, with a further 35 citations from grey literature, of which 20 met the inclusion criteria. These 20 citations reported on a total of 135 different case studies: 11 UK HIA; 11 non UK high income countries HIA, 5 UK SEA or other integrated appraisal; 108 non UK high income SEA or other integrated appraisal. All studies were in English. No relevant studies were identified reporting on low or middle income countries.

The studies were limited by potential bias (no independent evaluation, with those undertaking the appraisal also responsible for reporting outcomes), lack of detail and a lack of triangulation of results. Health impact assessments generally covered the four specified health domains (physical activity, mental health and wellbeing, environmental health issues such as pollution and noise, injury) more comprehensively than SEA or other integrated appraisals, although mental health and wellbeing was an underdeveloped area. There was no evidence available on the incorporation of health in Sustainability Appraisal, limited evidence that the recommendations from any type of appraisal were implemented, and almost no evidence that the recommendations had led to the anticipated outcomes or improvements in health postulated.

**Conclusion:**

Research is needed to assess (i) the degree to which statutory plan appraisal processes (SA in the UK) incorporate health; (ii) whether recommendations arising from health appraisal translate into the development process and (iii) whether outcomes are as anticipated.

## Introduction

Spatial planning affects the built environment, which in turn has the potential to have a significant impact on health, for good or ill For example, the level of active travel (walking and cycling) and outdoor recreational activity is strongly affected by accessibility to local facilities. Access to green, natural environments, and to local social networks, are factors in mental well-being. The wider sub-regional pattern of housing, economic development, land use and transport is a determinant of social exclusion and therefore health inequalities [1]. One way of ensuring that spatial plans take due account of health is through the inclusion of health considerations in the statutory and non statutory appraisal processes linked to planning processes. The appraisal of plans is a key statutory element of the plan-making process in most developed countries, running in principle, in parallel with the policy development process, helping to provide the rationale and evidence base for good decisions. Plan appraisal should be distinguished from project appraisal, which assesses the impact of specific development proposals.

Different appraisal and assessment techniques deal with health to different degrees; Health Impact Assessment (HIA) of course has health as its raison d'être, but is not a statutory requirement. In contrast, Strategic Environmental Assessment (SEA) and Sustainability Appraisal (SA) should, if properly undertaken, include consideration of all the main environmental determinants of health. (SEA) is a requirement in all countries in the European Union under the European Directive 2001/42/EC, and this assessment must consider both 'Human Health' and 'Population'. This has recently been extended with the Protocol on SEA to the United Nations Economic Commission for Europe (UNECE) Espoo Convention, of which the European Union is party, which came into force on 11th July 2010, and provides a legal basis for enhanced attention to human health in the SEA process, and for the health sector to to be routinely consulted on development plans [2]. SA and SEA are treated as one process in the UK. SA, even more than SEA, has the obligation of examining impacts on social variables, including health, well-being, quality of life and equity. Equality Impact Assessment (EqIA) is a process for identifying the potential impact of a project or land use policy, service and function on a population to ensure it reflects the needs of the whole community and minimise the potential for discrimination.

The study (commissioned by the National Institute of Clinical Excellence) [3] aimed to review the effectiveness of assessment and appraisal in terms of influencing planning decisions at the plan level to secure improvements in health and address health inequalities. The study took as its underpinning the assumption that development plans are likely to result in changes to the built environment that are then likely to influence health in a number of ways [1]. This will be primarily through changes in the patterns of determinants of health, which are then associated with changes in health outcomes. (It is important to note that this study has examined the impact of assessment and planning only in relation to spatial planning; health impact assessment and equality impact assessments are widely used in a variety of different arenas, including wider policy arenas which are not considered here).

## Methods

The search strategy to identify evidence from electronic databases was developed in an iterative manner to explore the concept areas of assessment/appraisal processes, plan initiatives and health outcomes (Additional file 1). A wide range of types of appraisal:Health impact assessment, Sustainability assessment, Strategic environmental assessment, Social impact assessment or appraisal, Integrated assessment or appraisal, Equity impact assessment or appraisal and Equality impact assessment or appraisal were included. Initial scoping of electronic databases suggested that Embase contained more relevant indexing terms than Medline, and therefore Embase was used to develop the initial search strategy that was subsequently adapted and applied to a further 13 electronic databases between November 2009 and January 2010. In addition a website searching protocol was applied to a selected list of UK and international websites. Bibliography lists of included studies were reviewed. Full details of the search strategy and terms are available [3].

Studies were included if all of the following criteria were met:

• the proposed plan would have an impact on human population

• the appraisal or assessment was undertaken as part of a regulatory process to examine the impact of the proposed plan

• there was an an objective evaluation of the impact of the appraisal as an intervention in time or in setting

• health issues were reported

• the full text was available in English

• published after 1987 (the publication of the Brundtland Report: Our Common Future, by the World Commission on Environment and Development)

No language restrictions were applied when conducting electronic database searches. All references identified were screening using title, abstracts or full texts, facilitated through the use of a checklist screening tool. Titles and abstracts of de-duplicated citations were screened independently by two reviewers to determine eligibility where adequate information was available. A data extraction form was developed for included studies to review the extent to which each study provided evidence on any/at least one of the following:

• Health issues were considered in the appraisal

• Health-related recommendations were incorporated into the plan

• Health-related recommendations were implemented

• Post plan adoption health outcomes were evaluated.

Four health issues were explicitly considered: physical activity, mental health and well being (including consideration of social networks), environmental health factors (air quality, noise pollution) and unintentional injury. If other specified potential impacts (such as employment or health equity) were described these were noted on the data extraction form. Data extraction was undertaken by a single reviewer who was not blind to the name of the authors, institution or source of the citation. Difficulties in data extraction were resolved through discussion within the review team.

### Assessing the quality of the evidence

To assess study quality each included paper was critically appraised using the methods developed by NICE [4]. An Internal validity score (to indicate potential sources of bias within the study) and an external validity score (to indicate the extent to which a study's findings may be considered generalisable to a wider population) were given to each included study.

Because of the differing regulatory frameworks within developed and less developed countries, and the particular interest of appraisal in the UK, the studies were grouped by UK, other high income countries and medium/low income countries.

## Results

**Figure 1:**
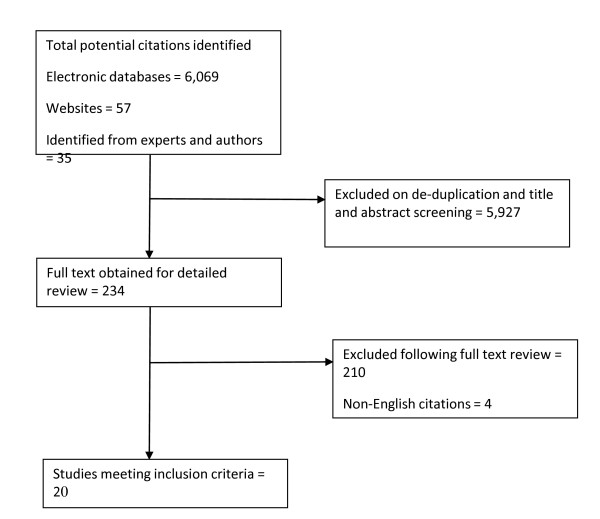
**Flowchart of included and excluded studies**.

A total of 6161 citations were identified: 6069 from the electronic databases, 57 from website searches, and afurther 35 citations were identified from grey literature, primarily a call for evidence by NICE on the topic of spatial planning and health. De-duplication, followed by screening of title and abstracts, excluded 5,926 citations. The full text of 234 remaining citations were obtained and screened. Of these, 20 met the inclusion criteria and quality checks (Figure 1). These 20 citations reported on a total of 135 different case studies: 11 UK HIA; 11 non UK high income countries HIA, 5 UK SEA or other integrated appraisal; 108 non UK high income SEA or other integrated appraisal. All studies were in English. (We were unable to access the full text of four potentially suitable articles with English abstracts but full text in other languages). No relevant studies were identified reporting on low or middle income countries. Some studies evaluated one case study while other evaluated multiple case studies, and some evaluated more than one type of appraisal.

Five of the 20 citations achieved [++] for external validity, with two citations only scoring [-]. The remainder scored [+], these were judged to be satisfactory either due to their use of publicly sourced documents and/or clear methodology, Limitations included potential bias (no independent evaluation, with those undertaking the appraisal also responsible for reporting outcomes), lack of detail and a lack of triangulation of results.

A summary of all included papers is shown in Table 1.

**Table 1:** Summary of all 'included' studies (Alphabetical order by first named author)

Study identification Author, year of publication	Country	Internal validity score ++/+/-	External validity score ++/+/-	Appraisal type	Subject of Appraisal
Corburn, J. & Bhatia, R. (2007)[5]	USA	+	++	HIA/IA	Urban housing redevelopment

Dannenberg, A., et al. (2008)[6]	USA	+	+	HIA	1. Rincon Hill Area Plan 2004 - Area plan for new downtown residential neighbourhood
					
					2. Eastern Community Neighbourhoods Community 2006
					
					3. City of Decatur community transportation plan 2007
					
					4. National petroleum reserve - Alaska - oil development plan, Alaska 2007
					
					5. Derby redevelopment 2007 Masterplan, zoning ordinance, design guidelines and budget request for community development project

Douglas, M., et al. (2001)[7]	UK	+	+	HIA	Draft Local Transport Strategy

Douglas, M., et al. (2007)[8]	UK	+	+	HIA	1. West Yorkshire Local Transport Plan (2000)
					
					2. City of Edinburgh Council's Urban Transport Strategy (2000)
					
					3. London Mayoral Strategy on Transport (2000)
					
					4. Thurrock Local Tranport Plan (2001)
					
					5. The 2003 West Midlands Local Transport Plan (2003)

Farhang, L, et al. (2008) [9]	USA	+	++	HIA	Rezoning plan for the Eastern Neighborhoods of San Francisco

Fischer, T., et al. (2009) [10]	UK/Germany	+	++	SA/SEA	1. Scoping Report and Core Strategy Preferred Options Report
					
					2. Local Transport Plan 2
				
				SA/SEA SA	3. Scoping Report and the Key Issues and Strategy Options for a Local Development Plan
					
					4. Regional plan of Western Saxony 2008
					
					5. Draft local statutory land use plan of Leipzig 2005
					
					6. Structure vision for Emmen

France, C. (2004)[11]	UK	+	+	HIA	Review of adopted Structure Plan policies and revision of emerging Structure Plan.

Glasgow Centre for Population Health (2007) [12]	UK	+	-	HIA	Draft Local Development Strategy

Gow, A., & Dubois, L. (2007) [13]	Australia	+	+	HIA	Two potential residential developments

Greig, S., et al. (2004) [14]	UK	+	+	HIA	Planning study of motorway corridor to inform a regeneration investment strategy

Kørnøv, L. (2009) [15]	Denmark	+	++	SEA	Review of 100 Danish SEAs

Ng, K., & Obbard, J. (2005) [16]	Hong Kong	+	+	SEA	Strategic planning case studies:
					
					1. territorial development strategy review
					
					2. third comprehensive transport study

Mathias, K., & Harris-Roxas, B. (2009) [17]	NZ	+	+	HIA	Greater Christchurch Urban development strategy

Mindell, J., et al. (2004) [18]	UK	+	+	HIA	Draft Transport Strategy

Neville, L., et al. (2005) [19]	Australia	+	+	HIA	Shellharbour Foreshore Management Plan, environment management plan with some land use issues

Planning Advisory Service (2008) [20]	UK	+	+	EqIA	Final draft masterplan to inform the Sustainability Appraisal of plan

Plant, P., et al. (2007) [21]	UK	+	-	IIA	Further Alterations to The London Plan

Stevenson, A., et al. (2007) [22]	NZ	+	+	HIA	Greater Christchurch Urban Development Strategy 2005

Tennant, K and Newman, C. (2007) [23]	Australia	+	+	HIA	Greater Granville Regeneration Strategy

Wismar, M., et al. (2007) [24]	UK, Finland, NL	+	++	HIA/SIA	1. Draft Air Quality Action Plan
					
					2. Detailed local plan for Korteniitty - complement an existing residential area with low and dense construction
					
					3. Plan for restructuring an industrial area into a residential area

### UK HIA

Seven citations were identified, reporting eleven case studies, from Scotland, England and Northern Ireland over the previous 10-12 years [7,8,11,12,14,18,24]. Transport plans/strategies are perhaps over-represented (seven of the eleven case studies).

Whilst there is comprehensive consideration of health issues in the plan appraisal, there is little reported evidence that this consideration led to changes in the plans themselves, nor that changes were implemented nor had an impact on health outcomes. Three report evidence of health recommendations being incorporated into the adopted plan [11,12,18], but only one case study [14] reported evidence of health recommendations being carried through into the implementation of the plan and of evaluation of the plan having been done. However, in two cases [11,24], the authors indicate that effectiveness could not be reported as the plan was not yet finalised. Alongside the four pre-specificed issues, others considered included community networks, access to health services, equity issues, the physical environment upgrade and community transport provision.

### Non UK high income countries HIA

Nine citations were identified that report 11 relevant case studies in four countries [5,6,9,13,17,19,22-24] in the USA, Australia, New Zealand and The Netherlands, although three studies [5,6,9] report on the same HIA for rezoning plan for the Eastern Neighbourhoods of San Francisco, and two studies [17,22] both report on an HIA for Greater Christchurch Urban Development Strategy 2005. Although the context for HIA in these different locations is somewhat different, in none of these countries is there a statutory duty for local authorities to undertake HIA, although differing levels of guidance are provided; in 2005, the New Zealand Public Health Advisory Committee issued guidance on HIA.

In eight of the 11 case studies, it was reported that health recommendations were incorporated into the plans, there is no clear evidence that health considerations influenced the implementation of the strategy, either because the citation did not report on it or the policy process was still not advanced enough at the time of writing to report on post adoption impacts.

Generally speaking, the case studies covered all the four specific health issues but only three case studies dealt with all the four specified issues. Ten covered other health outcomes, including access to services, urban design and housing, availability and control over housing, social connectedness, housing, transport, engagement with Maori, neighbourliness, and social cohesion were considered.

### UK: SEA and other integrated appraisals

Three citations were identified reporting five case studies [10,20,21]. Integrated appraisals are considered here with the UK SEA evidence as they aimed at informing the plan's SEA. A number of the UK SEA case studies were local transport plans, and the health issues explored are generally consistent with those normally considered in these plans, namely, increasing walking and cycling; reducing transport related pollution; reducing accidents, and reducing health inequalities by improving accessibility. The health issues considered for the two development plan documents at both the baseline and assessment stages were broad ranging, although mental health and wellbeing issues were addressed more indirectly, mostly through issues such as unemployment, lack of affordable housing, poverty, inequality, social exclusion and crime rates.

Whilst all case studies considered health issues in the appraisal process, it is unclear or not reported if health recommendations were incorporated into the plan, or whether the relevant policies were acted upon or implemented. No post plan impacts were reported although Plant [21] notes that key health indicators were to be included in monitoring the plan.

### Non-UK high income countries: SEA and other integrated appraisals

Three citations were identified that report 105 relevant case studies of SEA in four countries, including three detailed studies in Germany and the Netherlands, [10] an analysis of 100 environmental reports in SEA of 25 municipal plans and 75 local plans in Denmark, [15] of a wide variety of themes including housing, industrial areas, centre and leisure, transport and energy infrastructure, summer houses and golf courses, and of two detailed case studies in Hong Kong [16]. A further two citations were identified that report three relevant case studies from two countries [6,24] (Alaska, Finland) of integrated other types of appraisal.

The SEA Directive (Directive 2001/42) on the assessment of the effect of certain plans and programmes on the environment is implemented by all EU member states and serves as legislative basis for case studies in Germany, the Netherlands and Denmark. The Hong Kong Special Administrative Area's Government issued a circular in 1988 integrating environment assessment process consistent with SEA within the planning process of Hong Kong.

Whilst all citations provided evidence that health issues are considered in SEA, only one case study reported that health recommendations were incorporated into the plan - a transport study in Hong Kong [16]. Another study, a synthesis of 100 case studies was limited to examining the health issues *considered *in SEA and consequently did not report on how health considerations impacted on the specific plans [15]. None of the case studies provided evidence that the SEA health recommendations had been implemented at post adoption stage.

The range of health issues considered in the case studies varied, although only one refers to mental wellbeing, and the two studies from Hong Kong do not appear to report on any issues other than those relating to environmental health (air quality, water quality, accidents). Other issues included were light pollution, biodiversity, and risk of crime.

**Figure 2:**
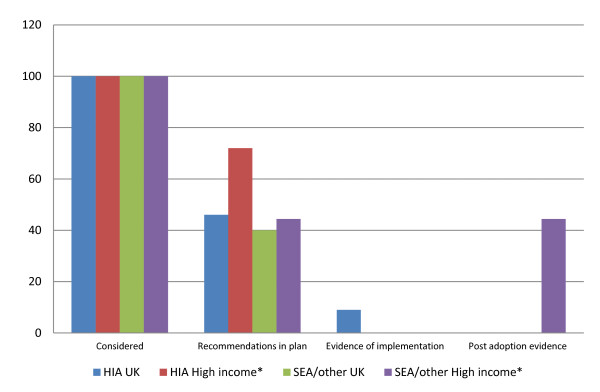
**Percentage of case studies reporting the extent to which health related recommendations were progressed *n *= 135 (note on report included 100 case studies)**.

**Figure 3:**
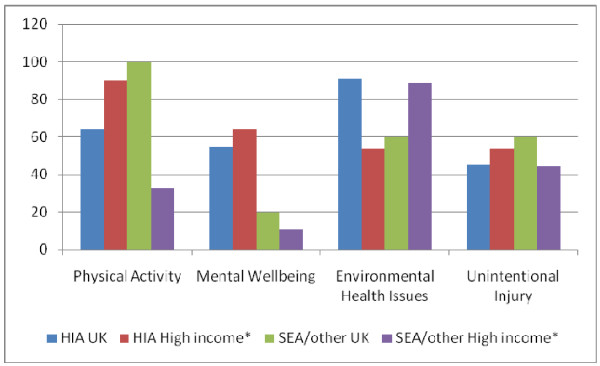
**Percentage of case studies reporting different health issues considered in appraisal (*n *= 135 case studies- note one report includes an overview of 100 case studies)**.

Figure 2 summarises the the extent to which the reports provided evidence within each of the 135 case studies that health issues were considered in the appraisal, were incorporated into the plan, were implemented, and whether post plan adoption health outcomes were evaluated. Figure 3 provides an overview of the areas of health that were reported as being considered within each of the 135 case studies. As only a high level summary of 100 appraisals was provided by Kørnøv [15], this was presented as one single case study for the purpose of both of these analysis. Results are reported by type of appraisal and by UK versus non UK, due to the differing requirements in different jurisdictions.

Figure 2 demonstrates that whilst there is ample evidence that health issues are considered, there is much less evidence demonstrating how this consideration translates into tangible recommendations in the plan making process, and even less about whether these are implemented and result in an impact on health. This deficit is particularly marked in the field of Health impact assessment in non UK countries, and SEA and other integrated appraisals in the UK.

Figure 3 demonstrates that whilst the four prescribed areas were reasonably covered, very few are consistently reporting across the wide breadth of health issues that might be expected. Mental health and wellbeing (including social wellbeing) was relatively infrequently reported, particularly in SEA and other integrated appraisals.

Equity issues, and consideration of the differential distribution of impacts appears to be relatively underdeveloped in all appraisals.Equity was mentioned explicitly as an important area that had been considered in the appraisal in six of the citations, three of which related to HIA [6,11,14,17,18,24].

## Discussion

Whilst SEA and SA are widely used and are statutory requirements across a wide range of juridstictions, there is a conspicuous lack of evidence of evaluations in this critical area relating to UK practice, with only three studies identified, and two relating to other forms of integrated appraisal (one an SA and one an EqA). Given the need public authorities to fulfil statutory equalities duties in the UK it is suprising that only one EqIA was identified.

There is little evidence that health issues were incorporated nor that health-related recommendations were incorporated into the adopted plan documents, and there is no information given about implementation. Whilst these case studies are highly applicable to the UK and the current spatial planning system, as only three case studies were identified, it is important to recognise that these examples may not be representative of SA/SEA practice in the UK. Outside the UK, there is strong evidence from all five case studies that health is considered in SEA, but no evidence that the SEA health recommendations had been implemented at post-adoption stage. One might argue, as Fishcher [10] has done, that, as the SEA directive requires that decision-makers should take the overall results of the assessment into account it is "probable" that health considerations had an impact, but were unable to identify little empirical evidence to support this assumption.

Similar issues in terms of evaluation are found in relation to HIA. Of the eleven UK case studies identified, only one case study reported HIA effectiveness in terms of completion of all stages from health recommendations, to implementation and post adoption evaluation [14]. Many reported that those involved felt the process was useful, indeed successful, in improving the plans, and (in some cases) empowering local communities and environmental interests. Keys to success were seeing the HIA as part of an iterative process throughout plan preparation, and the active involvement of planners with health and other professionals. The evidence from HIA of plans in non UK high income countries suggests that the HIAs generally influenced the plan, although the degree of that influence is varied, even contested, with some analysts suggesting it is more often through raised health awareness of the decision-makers than directly as a result of the assessment.

The case studies strongly suggest that factors such as the timing of appraisal (late HIAs have been reported to have limited impact), and community engagement are critical in the success of appraisal. Full integration of comprehensive health assessment into existing formal and statutory processes increase the likelihood of health being properly considered and incorporated into the plan. However, there is a lack of data on outcomes to support this supposition.

There are limitations in the literature reviewed. Many of the publications are reports from authors who have themselves been directly responsible for undertaking the appraisal, with little independent evaluation or triangulation of reported findings, thus leading to potential bias. We were unable to access the full text of four potentially suitable articles with English abstracts but full text in other languages. Given the complexity and timescale for development, there are practical difficulties in both tracking, and attributing recommendations and changes in plans and subsequent developments to appraisal processes. Whilst the lack of evidence per se does not mean that there is a lack of effectiveness, the dearth of evidence linking appraisals to implementation and subsequent changes in outcomes is challenging. Concerns about the lack of evaluation of the impact of HIA have also been noted in the past by others [25], and guidance from Breeze and Lock [26] in 2001 highlighted the need to monitor impacts, record results of HIA, and to consider the need for monitoring of any anticpated impact(s) on people's health, but this seems to have had little impact. This may reflect the current lack of regulatory and financial requirements to carry out such evaluation, a limitation of the current development and planning processes, which are much more orientated to appraisal processes, often conducted by external consultants on a short term contract basis, who have no ongoing input at the implementation stage of the development. There is a clear case now for post hoc analysis of existing appraisals which would provide an opportunity to explore if predicted outcomes, for example on physical activity or mental wellbeing did actually materialise. There is also a case to be made to increase the emphasis on post-development monitoring, and to link appraisals more explicitly to outcomes. Whilst there are of course, significant difficulties in attributing any changes in health outcomes observed by post development monitoring, and in particular in attributing changes to either the appraisal itself, or the resultant changes in the built environment, further work in this area would enhance our understanding of the links between the built environment and health, and could inform further appraisals. Another useful focus for research might be to look at how and why health recommendations are implemented.

The study suggests that there is considerable variation in the degree to which health issues are comprehensively considered, with evidence that mental health and wellbeing issues may be particularly under-reported in SEA and other integrated appraisals. Equity issues, and consideration of the differential distribution of impacts appears to be relatively underdeveloped in all appraisals. This has implications for the training of those involved in undertaking appraisals. It is possible, that particularly during HIA, a fuller more comprehensive range of health issues was considered at the scoping and screening stage, but if no significant impacts were identified that these were not considered further.

Posas summarises the development of HIA in the context of SEA [2], highlighting that although health was not generally well considered in SEAs in the late 1990s and early 2000's, this began to change with the EU SEA directive (EC42/2001) with a statutory requirement for consideration of significant impacts on health as part of the EU process. This was facilitated in England, by the issuing of a consultation on draft guidance on health in strategic environmental assessment by the Department of Health [27], which it is anticipated will be re-issued in the near future. With the Protocol on SEA to the United Nations Economic Commission for Europe (UNECE) Espoo Convention coming into force on the 11 July 2010, there is a a legal basis for enhanced attention to human health in the SEA process. This provides a significant opportunity to have a more comprehensive approach to assessing health, and incorporating the use of HIA in informing SA/SEA processes.

However, health appraisal is only one of part of the development plan process; health considerations need to be built in at the very early conception and development of plans (arguably no additional health recommendations would be needed following appraisal of a totally robust plan), and critically, followed through to the development management process. There are clear implications for the training of planners, developers, and those involved in undertaking appraisals.

A particular point of note is the dearth of evidence from low and middle income countries. Outside the EU some countries have adopted SEA practice, or some strategic form of EIA, but there is very variable uptake and use of HIA as highlighted by Erlanger [28] who in a review of 237 HIA publications found only 6% had a focus on the developing world. Given the rapid scale of development in middle and low income countries and the variable development in planning legislation and environmental assessment, this is of concern.

In conclusion, action is required; firstly, to ensure that a firstly that a comprehensive approach to examining potential health impacts is undertaken, ensuring that relatively neglected areas such as mental health and well being and equity are addressed; secondly that due attention is paid to ensuring that the recommendations arising from consideration of health issues in stand alone or integrated appraisals are embedded into plans; thirdly that attention needs to be given to the current regulatory framework to ensure that evaluation and post-development monitoring is undertaken; and finally that there is more work undertaken to ensure that recommendations translate into the development process and that outcomes are as anticipated.

## Competing interests

No financial competing interest declared

Hugh Barton is Director of the WHO Centre for Healthy Urban Environments, UWE.

## Authors' contributions

SG and HB conceived the concepts; JM developed the search strategy and data screening and extraction tools; JJ, LM and HL undertook searching of electronic databases, screening and data extraction; SG, HL, JM and HB wrote project report and SG prepared paper for publication. All authors have commented on and approved of final draft.

## Sources of funding

NICE Centre for Public Health Excellence.

## Disclaimer

The views expressed in this paper are those of the authors and do not represent the views of NICE as funders of this work.

## Pre-publication history

The pre-publication history for this paper can be accessed here:

http://www.biomedcentral.com/1471-2458/11/889/prepub

## Supplementary Material

Additional file 1 **The effectiveness of health appraisal processes currently in addressing health and wellbeing during spatial plan appraisal: a systematic review**. Click here for file
