# Supplementary material for: The effectiveness of health appraisal processes currently in addressing health and wellbeing during spatial plan appraisal: a systematic review
Source: BMC Public Health. 2011 Nov 24;11:889. doi: 10.1186/1471-2458-11-889 (PMC3276579; doi:10.1186/1471-2458-11-889)
Supplement: Additional file 1 — The effectiveness of health appraisal processes currently in addressing health and wellbeing during spatial plan appraisal: a systematic review. [file 1471-2458-11-889-S1.DOC]

The effectiveness of health appraisal processes currently in addressing health and wellbeing during spatial plan appraisal: a systematic review

The Search Strategy details and outputs are appended here for convenience as a supplementary file: these can be accessed in file University of West of England (2011). The effectiveness and cost effectiveness of health appraisal processes currently in use to address health and wellbeing during plan appraisal. Report prepared for the National Institute for Health and Clinical Excellence (NICE). [**www.nice.org.uk**](http://www.nice.org.uk/). London: NICE.

**Questions that will be addressed**

**Appraisal approaches**

Q1 How effective are approaches to appraisal in terms of influencing planning decisions (at the plan and project level) to secure improvements in health and address health inequalities?

Q2 What lessons can be learnt from other countries about the effectiveness of the above approaches?

**Equity**

Q3 What is the evidence that health equity issues are effectively considered as part of the appraisal of spatial planning decision-making processes? 89

**Search approach and rationale**

The search approach taken will be systematic, but the review team acknowledge that the ability to apply the standard methods for the development of NICE public health guidance to a distal determinant of health such as spatial planning may be constrained. Limitations may arise due to the bringing together of two disciplines (spatial planning and health) with differing definitions, evaluative methodologies and levels of evidence of effectiveness available.

The review team propose that the search strategy undertaken for reviews 1 and 2 will be identical and that identification of studies meeting the inclusion criteria for review 1 (project appraisal) and those meeting the inclusion criteria for review 2 (plan appraisal) will be differentiated during the screening of titles and abstracts, and will be facilitated through the use of a screening tool, as recommended by the NICE Technical Lead. The screening tool will be a checklist for the reviewer screening the titles and abstracts to confirm whether the paper does, or does not, meet the inclusion criteria for review 1 (project appraisal) or review 2 (plan appraisal).

Scoping of databases and search terms indicate that searches will need to be primarily sensitive (to identify relevant information) rather than specific (exclusion of irrelevant material) due to the limited use of indexing and coding terms for the subject areas of spatial planning and assessment / appraisal. The review team propose that EMBASE be used to develop the initial search strategy because the early scoping of the databases suggested that although neither Medline nor Embase contains particularly helpful indexing terms for spatial planning, Embase contained more relevant subject headings than Medline. This search strategy will then be adapted for each of the other databases listed, as appropriate. The clinical databases are much more limited in the availability of relevant subject headings than the non-clinical databases, and the latter are likely to allow a greater degree of precision within the search history than in the clinical databases. 90

**Key words and concepts**

We anticipate that the search strategy will focus on 2 main concepts:

Concept 1: Appraisal and assessment processes

To include key words / subject headings that cover

Tools: „Impact assessment‟ (all types)

„Appraisal‟ (all types)

Specific policies: Regional spatial strategy

Local development frameworks

Local transport plans

Regeneration strategies

Concept 2: Health outcomes

To include key words / subject headings that cover

Health (broadest definition)

Specific outcomes: Physical Activity

Mental health and wellbeing

Healthy environment (e.g. air quality)

Unintentional injury

Practitioners and communities engagement with health issues

**Electronic sources that will be searched**

1. Core databases

EMBASE

MEDLINE

HMIC

PsycINFO

Cochrane Database of Systematic Reviews

Cochrane Central Register of Controlled Trials

Database of Abstracts of Reviews of Effectiveness (DARE)

Social Science Citation Index

2. Additional databases

GEOBASE

PLANEX

91

Transport Research Information Systems (TRIS) and / or Transport

ICONDA

URBADOC

CAB Abstracts

3. Websites

We suggest focusing on those websites that directly consider impact assessment. Websites under consideration to search for reports and documents that meet our inclusion criteria include:

NICE

HDA publications (via www.nice.org.uk/page.aspx?o=hda.publications)

UK and Eire Public Health Observatories

Department for Transport

Department of Communities and Local Government

Department for Environment, Food and Rural Affairs (DEFRA)

Planning Inspectorate

Royal Town Planning Institute (RTPI)

Chartered Institute of Environmental Health (CIEH)

WHO (Healthy Cities)

Commission for Architecture and the Built Environment (CABE)

International Association for Impact Assessment

Resource for Urban Design Information (RUDI)

ISURV

Planning Advisory Service

VicHealth

International Health Impact Consortium

American Planning Association

Town and Country Planning Association

ICLEI

Environment Agency

Natural England

Scottish HIA Network

92

**Grey literature**

Grey literature sources are likely to be particularly valuable as the limited coding and indexing terms for spatial planning and appraisal / assessment may restrict the number of studies identified from electronic databases. Expert and author contacts will be made requesting both (i) articles known to meet our inclusion criteria and (ii) review articles on the value of appraisal / assessment of plans and projects in health improvement. Bibliography lists of such reviews may indicate studies meeting the inclusion criteria.

Follow up of grey literature sources whilst valuable, are time-consuming, and therefore may need to be limited. Grey literature sources will therefore include:

Bibliography lists of included studies

Bibliography lists of review articles suggested by experts and authors

Follow up of references that may meet inclusion criteria suggested by experts and authors in the field

**Use of a screening tool**

Results of the electronic database searches will be downloaded to a reference management software tool; RefWorks. Within RefWorks the results of each electronic database will be filed separately. Sources that cannot be automatically downloaded will be viewed on screen to identify those that meet the inclusion criteria and these will be manually entered into their own file in RefWorks. Numbers of citations retrieved and excluded from non-downloadable databases will be documented. In RefWorks a duplicates search will be run to allow duplicates to be identified and excluded. Titles and abstracts of de-duplicated citations will be viewed on screen to determine whether or not they meet the inclusion criteria using a screening tool that will determine eligibility for either review 1 or review 2. At this stage articles that may be interesting for the context, methodology, author expertise or relevance to later reviews will also be identified and catalogued. 93

**Inclusion and exclusion criteria**

**a) Inclusion criteria**

4. Population

The human population affected by the proposed project or plan (reviews 1 & 2)

5. Intervention

The appraisal or assessment of the impact of the proposed project (review 1) or plan (review 2) on the health of the local population.

Technologies and tools to conduct such appraisals include but are not limited to; Strategic Environmental Assessment (SEA), Sustainability Appraisal (SA), Environmental Impact Assessment (EIA), Health Impact Assessment (HIA), Sustainability Impact Assessment (SIA), Integrated Appraisal, Social Impact Assessment (SIA), Equity Impact Assessment, Inequality Impact Assessment, (reviews 1 & 2).

Projects and plans may also be referred to using a variety of other terms including but not limited to; strategies or frameworks, which will specifically include Regional Spatial Strategies, Local Development Frameworks, Local Transport Plans (reviews 1 & 2)

6. Comparison

No use of the appraisal or assessment process e.g. before and after studies (reviews 1 & 2)

An alternative appraisal or assessment process e.g. between country studies (reviews 1 & 2)

7. Outcomes

One or more of the following outcomes (reviews 1 & 2)

Were health outcomes (including health equity issues) considered in the appraisal / assessment process?

Were any specific recommendations about health outcomes included following appraisal / assessment?

94

Were health recommendations acted upon? / Was there any evidence that any of the health recommendations were implemented?

Was there any evidence of an impact on health? Specifically:

o Changes in levels of physical activity?

o Mental health and wellbeing?

o Environmental issues affecting health (including air, water & noise pollution, contaminated land, waste management)

o Unintentional injury?

Knowledge and skills of planners of the importance of health outcomes?

Was there evidence of participation and engagement of communities / populations / stakeholders in the discussion of health outcomes?

Examples of study types that will be included (reviews 1 & 2)

Before and after studies

Ecological studies

Case-control or case-comparison studies

Evaluated case reports or case series

Note: The review team considers it unlikely that evidence from study designs towards the top of the hierarchy of evidence (e.g. RCTs, controlled non-randomised trials, etc) will be found

**Restrictions on searches**

3. Time period

Studies conducted since 1987 (publication of the Brundtland Report: Our Common Future, by the World Commission on Environment and Development)

4. Language

No language restrictions will be applied at the search stage of reviews 1 & 2 for electronic database searches.

95

We acknowledge that this is contrary to the standard methods for the development of NICE public health guidance but is proposed for two reasons:

1. The review team is aware of good practice in other countries (principally European and Scandinavian countries) that may not be published in English

2. To competently answer Q2 it is necessary to include non-English language articles at the search stage to be able to identify potentially valuable papers.

It is proposed that, as the majority of non-English language articles will include an English translation of the title and abstract, all languages should be included in the electronic database searches to allow quantification of the contribution of non-English literature to the evidence base. Discussion with NICE will determine subsequent decision-making on how to manage / document these non-English language papers e.g an appendix may report the English titles and abstracts of these papers should we chose to exclude them.

Spatial Planning for Health Collaborating Centre

23rd November 2009 96

**Appendix B: Search methodology and strategy**

The search strategy applied to electronic databases is detailed below; this strategy was adapted to accommodate searching of the other databases, some of which did not allow the ease or flexibility afforded by Embase.

| **Embase (1980 to 2009 Week 50)** 1 | (spatial or structur$ or core or urban$ or rural or municipal$ or town$ or settlement$ or village$ or region$ or sub-region$ or subregion$ or city or cities or neighbourhood$ or neighborhood$ or local$ or suburb$).tw. | 1978715 |
| --- | --- | --- |
| 2 | exp urban area/ or exp rural area/ or exp suburban area/ or exp city/ | 37536 |
| 3 | (sustainab$ or environment$ or economic$ or social or conservat$ or landscape$ or accessib$ or regenerat$ or renewal or redevelop$).tw. | 666087 |
| 4 | exp environment/ or exp landscape/ | 1768262 |
| 5 | (transport$ or cycl$ or bicycl$ or pedestrian$ or walk$ or non-motori#ed or road$ or ringroad$ or rail$ or tram$ or bridge$ or tunnel$ or train$ or underground or metro$ or tube or TGV or motorway$ or street$ or autobahn$ or freeway$ or expressway$ or autostrada or turnpike$ or super#highway$ or carriageway$ or highway$ or path$ or link$ or bus or buses or coach$ or route$ or interchange$ or bypass$ or airport$ or heliport$ or port$ or terminal$ or harbour$ or harbor$ or cargo$).tw. | 2717494 |
| 6 | exp motor vehicle/ or exp bicycle/ or exp motorized transport/ or exp pedestrian/ or exp walking/ or exp railway/ or exp airport/ | 41910 |
| 7 | (active adj travel).tw. | 18 |
| 8 | ((open or recreation$ or leisure or commun$ or public or play or green or blue) adj space$).tw. | 526 |
| 9 | (park$ or recreation$ or leisure or greenspace$ or garden$ or playground$).tw. | 73550 |
| 10 | exp recreation/ or exp leisure/ | 13595 |
| 11 | ((land or single or mixed or multi) adj "use").tw. | 4152 |
| 12 | (shop$ or retail$ or outlet$ or market$ or supermarket$ or mall$ or arcade$ or wholesale$ or business$ or office$ or industr$ or commerc$ or service$ or school$ or college$ or universit$ or hospital$ or clinic$ or surger$ or infrastructur$ or building$).tw. | 2662130 |
| 13 | (quarr$ or excavation$ or mine$ or dredg$).tw. | 77384 |
| 14 | ((holiday or chalet or caravan) adj (park$ or camp$ or site$ or village$)).tw. | 37 |
| 15 | (mast$ or pylon$ or pipeline$ or (overhead adj cable$)).tw. | 62690 |
| 16 | (hydro#electric$ or nuclear or coal or gas or oil or fuel or electricity).tw. | 387496 |
| 17 | renewable energy.tw. | 291 |
| 18 | exp commerce/ or exp business/ or exp school/ or exp college/ or exp university/ or exp hospital/ or exp health center/ | 244777 |
| 19 | ((scienc$ or techno$ or educat$ or health) adj park$).tw. | 32 |
| 20 | ((distribution or communit$ or health or leisure) adj (centre$ or center$)).tw. | 8877 |
| 21 | (river$ or water or reservoir$ or canal$ or coast$ or fluvial or pluvial or flood$ or swale$ or drain$ or rain$).tw. | 437721 |
| 22 | exp river/ or exp water management/ or exp flooding/ or exp seashore/ or exp rain/ | 94307 |
| 23 | (home$ or residen$ or accommodat$ or estate$ or hous$ or apartment$ or flat$ or condominium$).tw. | 333491 |
| 24 | exp home/ or exp housing/ or exp accommodation/ or exp residential area/ | 13036 |
| 25 | (incinerat$ or landfill$ or waste or recycl$ or compost$).tw. | 53478 |
| 26 | exp landfill/ or exp recycling/ or exp incineration/ or exp waste management/ or exp composting/ | 82108 |
| 27 | ((air or water or noise or land or soil) adj (quality or pollut$ or contaminat$ or protect$ or prevent$)).tw. | 30227 |
| 28 | exp air quality/ or exp air pollution/ or exp water quality/ or exp water pollution/ or exp noise pollution/ or exp soil pollution/ | 144654 |
| 29 | (eco#town$ or eco#village$).tw. | 0 |
